# Ophthalmological manifestations of the Schuurs-Hoeijmakers syndrome: a case report

**DOI:** 10.5935/0004-2749.20220013

**Published:** 2022

**Authors:** Mariana W. de Barros e Silva, Alline Martins, Analine L. de Medeiros, Adriano de Vasconcelos, Camila V. Ventura

**Affiliations:** 1 Centro Universitário de João Pessoa, João Pessoa, PB, Brazil; 2 Department of Ophthalmology, Fundação Altino Ventura, Recife, PE, Brazil; 3 Department of Ophthalmology, Hospital de Olhos de Pernambuco, Recife, PE, Brazil

**Keywords:** Microphthalmos, Optic nerve, Eye manifestation, Abnormality, Multiple/genetics, Phenotype, Syndrome, Developmental disability, Microftalmia, Nervo óptico, Manifestação ocular, Anormalidade múltipla/genética, Fenótipo, Síndrome, Deficiência do desenvolvimento

## Abstract

This is a case report of a 2-year-old male patient with cognitive delay, facial abnormalities, and microcornea in the right eye, who was referred for ophthalmological investigation. The initial ophthalmological examination revealed hypertelorism, epicanthus, nystagmus, esotropia, and microcornea in the right eye. The examination under anesthesia revealed microphthalmia in the right eye, and iris, retina, and optic nerve coloboma in both eyes. Whole exome sequencing revealed evidence of a heterozygotic pathogenic variant in *PACS1*. The *PACS1* pathogenic variant in association with the clinical findings confirmed the diagnosis of Schuurs-Hoeijmakers syndrome. To our knowledge, this is the first report to describe microcornea and microphthalmia as additional ocular manifestations of Schuurs-Hoeijmakers syndrome.

## Abstract (pt)

Trata-se de um relato de caso de um paciente do sexo masculino de 2 anos de idade com atraso cognitivo, anormalidades faciais e microcórnea no olho direito encaminhado para investigação oftalmológica. O exame oftalmológico inicial evidenciou hipertelorismo, epicanto, nistagmo, esotropia e microcórnea no olho direito. O exame sob anestesia revelou microftalmia no olho direito e coloboma de íris, retina e nervo óptico em ambos os olhos. Foi realizado sequenciamento completo do exoma que mostrou uma variante patogênica heterozigótica no *PACS1*. A variante patogênica no *PACS1* em associação com os achados clínicos apresentados confirmou o diagnóstico da síndrome de Schuurs-Hoeijmakers). Acredita-se este seja o estudo a descrever microcórnea e microftalmia como manifestações oculares adicionais da síndrome de Schuurs-Hoeijmakers.

## INTRODUCTION

Schuurs-Hoeijmakers syndrome (SHS) or *PACS1*rela ted syndrome is rare and was first reported by Schuurs-Hoeijmakers in 2012 in patients from the Netherlands and Belgium^(1)^. Two years later, the same mutation was reported in a German child with similar clinical findings^(2)^. In 2016, the largest sample with 19 patients with SHS was described by Schuurs-Hoeijmakers et al.^(3)^. All the patients were affected randomly and had no other family history of SHS.

The initial report revealed that SHS is caused by a heterozygote pathogenic variant in the *PACS1* gene, which, according to the results of animal studies, leads to the substitution of amino acids (arginine for tryptophan) in the furin-binding region of the protein and exerts a negative dominant effect^(1)^.

The pathophysiology of the *PACS1* protein mutation, studied in vitro and in vivo, showed that this gene is essential for the formation of craniofacial structures, and its dysfunction leads to neural crest migration deficiency. Therefore, the defective function of the *PACS1* protein can cause intellectual delay, abnormal facial features, and ophthalmic conditions^(1,3)^.

The facial changes include highly arched eyebrows, long eyelashes, hypertelorism, down-slanting palpebral fissures, ptosis, rounded nasal tip, wide mouth with corners that point downward, widely spaced teeth, and low-set ears^(1,3,4)^. With regard to the ophthalmological manifestations of SHS, nystagmus, strabismus, and coloboma of the iris, optic nerve, and retina were described^(3,4)^. In addition, owing to the *PACS1* pathogenic variant, other physical changes may occur, including heart and brain malformations, and cryptorchidism in male patients^(3)^.

Although a diversity of ocular findings has been described in association with SHS, to our knowledge, this is the first report to describe microcornea and microphthalmia in association with SHS. In addition, all other cases previously described were from Europe and Asia. Thus, we describe the first reported SHS case in the Americas.

## CASE REPORT

A 2-year-old patient was referred to our pediatric ophthalmology department for further assessment and investigation. The mother reported asymmetry of the child’s eyes and nystagmus. The child’s past medical history included intellectual delay. The mother denied any similar cases in the family.

**Figure 1:**
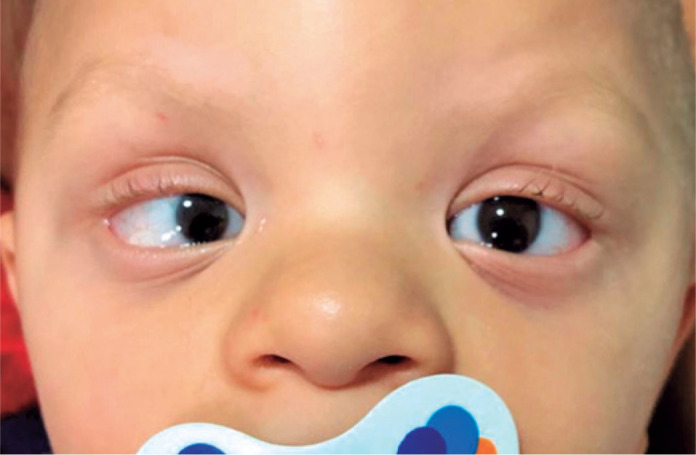
Low ear lobe implantation, hypertelorism, epicanthus, microcornea in the right eye, and round nasal tip in the child with Schuurs-Hoeijmakers syndrome.

Ectoscopy findings included low ear lobe implantation, hypertelorism (intercanthal distance of 30 cm), epicanthus, and rounded nasal tip (Figure 1). Ocular motility evaluation using the Krimsky method revealed esotropia of 16 prismatic diopters and horizontal nystagmus.

**Figure 2:**
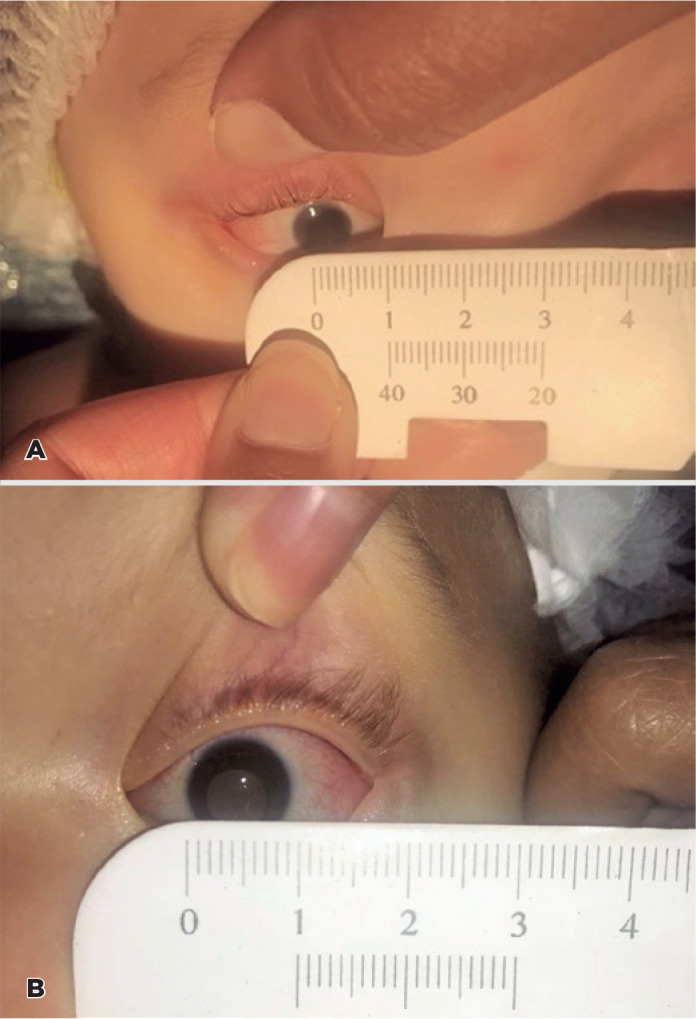
Corneal diameters of the right eye measuring 7 mm (A) and (B) left eye measuring 10 mm.

The patient’s best corrected visual acuity measured using Teller’s visual acuity cards showed high contrast (0.23 cy/cm at 83 cm) in the oculus dexter (OD) and 20/270 (3.2 cy/cm at 83 cm) in the oculus sinister (OS). The patient was examined under sedation for further investigation. The cycloplegic refraction was 13.00 DE in the OD, and -2.25 and -0.50 at 60° in the OS. The intraocular pressure (Perkins) was 8 mmHg in the OD and 14 mmHg in the OS. The corneal white-to-white measurement was 7 mm in the OD and 10 mm in the OS, indicating a microcornea in the OD (Figure 2).

**Figure 3:**
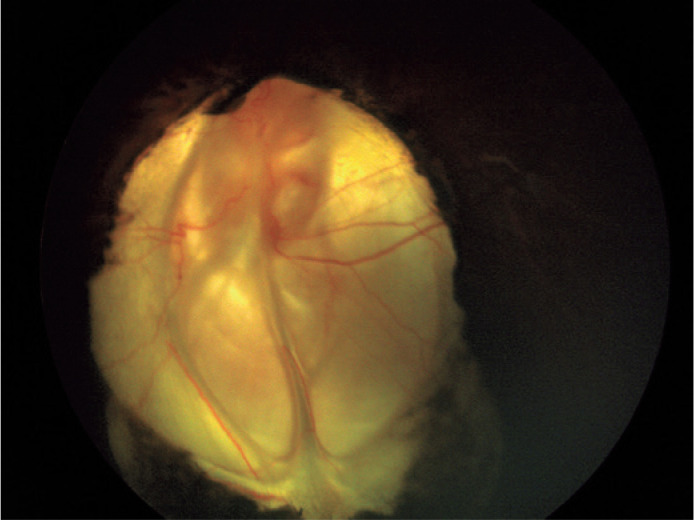
Color fundus image of the left eye showing the coloboma of the optic nerve and retina.

The anterior segment examinations revealed colobomas of the iris, optic nerve, and retina in the inferior quadrant of the oculus uterque (Figure 3). Ocular ultrasonography revealed no retrobulbar cyst, and the axial length was 20 mm in the OD and 22 mm in the OS.

The patient underwent complete exome sequencing, which revealed a heterozygotic pathogenic variant in the *PACS1* gene *(NM_018026.3:c.607C>T; p.Arg203TTrp in Chr11:6597867)*. Confirmation of the *PACS1* pathogenic variant in addition to the clinical manifestations such as intellectual delay, hypertelorism, and colobomas of the iris, optic nerve, and retina confirmed the diagnosis of SHS.

## DISCUSSION

We present the case of a 2-year-old Brazilian boy with SHS. Although this syndrome has been previously reported in the Netherlands, Germany, India, and Japan, to our knowledge, this is the first SHS case reported in the Americas^(1-5)^.

As in the previously reported cases, our patient presented the typical manifestations of SHS, including intellectual delay and facial features such as low ear lobe implantation, rounded nasal tip, hypertelorism, and epicanthus^(1,3-5)^. Moreover, our child presented the well-known ocular manifestations of this syndrome, including nystagmus, strabismus, and colobomas of the iris, optic nerve, and retina^(1,3)^. Nevertheless, in addition to these findings, our child also presented unilateral microcornea and microphthalmia. These findings were responsible for the significant anisometropia and amblyopia identified in the child and have not been previously reported in the literature in association with SHS.

The retina and optic nerve colobomas herein described have a wide range of differential diagnosis, including Walker-Warburg, CHARGE (coloboma, heart anomaly, choanal atresia, retardation, and genital and ear anomalies), Aicardi, and Knobloch syndromes^(6)^. Therefore, genetic investigation is crucial for proper management and diagnosis of these cases. In addition, detection of the optic nerve and retinal colobomas is important to determine their relationships with retinal detachment, as up to 60% of these eyes have been estimated to progress to retinal detachment by the time patients reach the age of 30-40 years^(6,7)^. This occurs when a break is formed at the margins of the coloboma that causes the vitreous to access the subretinal space and detach the retina.

Studies have also shown that retrobulbar cysts, present in 30% of optic nerve colobomas, increase the risk of retinal detachment^(8)^. Although our patient did not present a retrobulbar cyst, the presence of retinal and optic nerve colobomas raised a concern warranting close monitoring.

To our knowledge, this is the first case report to describe microcornea and microphthalmia as novel findings of SHS and a SHS case in the Americas. This study provides awareness of the risk of amblyopia in SHS and suggests close monitoring of patients for early detection of possible retinal detachments.
